# Exploring the relationship between problematic social networking sites use and depression: A longitudinal study

**DOI:** 10.1371/journal.pone.0313362

**Published:** 2024-11-18

**Authors:** Xiaoling Li, Hailei Li

**Affiliations:** 1 School of Psychology, Shandong Normal University, Jinan, Shandong, China; 2 School of Psychiatry, North Sichuan Medical College, Nanchong, Sichuan, China; 3 Business School, Shandong Normal University, Jinan, Shandong, China; Johns Hopkins Bloomberg School of Public Health: Johns Hopkins University Bloomberg School of Public Health, UNITED STATES OF AMERICA

## Abstract

The association between problematic social networking sites use and depression is established, yet the directionality remains to be clarified. This longitudinal study investigated the causal dynamics between the two phenomena by administering a questionnaire to 191 college students from a university in Shandong Province, China, across four assessment points with an interlude of four weeks. The study found that the impact of depression on problematic social networking sites use is not consistently sustained throughout the academic semester. Initially, this impact reached significance, but it waned and ceased to be significant during the mid- and late-semester phases. These findings allude to the possibility that depression acts as a significant precursor to problematic social networking sites use. However, the interaction between them appears to be subject to temporal and contextual shifts. Consequently, interventions tackling problematic social networking sites use should progressively pivot from moderating screen time and social media engagement towards enhancing emotional robustness.

## 1 Introduction

As the internet and smartphone technologies continue to evolve, the world becomes increasingly interconnected, and these devices have become indispensable parts of our daily lives. However, the integration of digital technology into every aspect of human existence has given rise to a concerning phenomenon: the problematic overuse of these technologies [1,2]. Within this broader context, two distinct patterns of problematic use have been identified: generalized problematic smartphone/internet use and specific problematic smartphone/internet use [3]. The former encompasses a wide-ranging and general overuse of smartphones or the internet, potentially leading to various negative impacts on individuals [4]. The latter, specific problematic smartphone/internet use, involves excessive engagement with particular online activities such as social media, gaming, or gambling, and has been linked to poor health outcomes [5–8]. Studies have found the prevalence rates of various behavioral addictions to be as follows: internet addiction at 10.6%, smartphone addiction at 30.7%, gaming addiction at 5.3%, and social media addiction at 15.1% [9].

Studying problematic social networking sites use (SNSU) is crucial for several reasons. Firstly, the nature of social networking sites engagement differs from online gaming, which typically has clearer start and end points. Social networking sites, on the other hand, involve continuous and dynamic social interactions that require significant emotional investment [10]. Moreover, research has revealed concerning prevalence rates for various forms of behavioral addictions, with social media addiction standing at 15.1% [9]—a significant portion of the population that may be adversely affected by their digital habits. Therefore, understanding and addressing problematic SNSU is essential for promoting healthier digital lifestyles and mitigating the potential harm associated with excessive and unregulated use of these platforms.

The rapid proliferation of social networking sites has revolutionized communication, providing users with unprecedented opportunities for social interaction, information exchange, and entertainment [11,12]. However, alongside these benefits, there is a growing concern about the potential negative consequences associated with excessive or problematic use of these platforms. Specifically, there is increasing evidence suggesting a link between problematic SNSU and adverse mental health outcomes, particularly depression [13,14]. Problematic SNSU is defined as an extreme focus on social networks, driven by a strong urge to log on or use social networks, using considerable time and energy on these platforms, thereby deteriorating other social activities, learning/work, interpersonal relationships, and/or mental health and happiness [13].

Depression, a prevalent mental health disorder, manifests as persistent feelings of sadness, loss of interest or pleasure in activities, and a variety of emotional and physical problems [15,16]. There is a close link between problematic SNSU and depression [17–19], but the direction of this relationship is complex. Longitudinal studies have found that problematic SNSU can significantly predict depression [20–23]. This conclusion is also supported by experimental research, which found that limiting daily SNSU reduces depression levels in college students [24]. However, other longitudinal studies have found that psychological distress at Time 1 can significantly predict increased levels of problematic SNSU at Time 2, but the reverse predictive relationship does not hold [25]. Gámez-Guadix [26] found that depressive symptoms at Time 1 significantly predict a preference for online social interactions at Time 2. Other longitudinal studies have found a bidirectional predictive relationship between problematic SNSU and depression [27]. However, the study by Li et al. [27] was conducted using two sub-samples, one examining problematic SNSU’s prediction of depression and the other exploring depression’s prediction of problematic SNSU, inevitably affecting the result’s reliability.

Despite the growing body of literature on the relationship between SNSU and psychological well-being, significant gaps remain in the research. Notably, there is a scarcity of studies employing a longitudinal design to examine these relationships within an East-Asian context [3]. Research on the link between depressive symptoms and SNSU has predominantly utilized cross-sectional methodologies, which limit the ability to infer causality [28]. Furthermore, most longitudinal studies have focused on adolescent populations, leaving a gap in understanding the causality between problematic SNSU and psychological distress among university students [21]. Addressing these limitations, the current study aims to explore the longitudinal relationship between problematic SNSU and depression among university students, thereby contributing to the existing literature by providing insights specific to an East-Asian university student sample.

According to the theory of compensatory internet use [29], internet addiction stems from individuals’ responses to negative life situations, and internet applications facilitate this response. For example, when individuals lack social stimulation in real life, they may turn to online social interactions, which can be facilitated by social networking sites. This can have both positive and negative consequences: on the positive side, individuals can feel uplifted by the social stimulation they receive; on the negative side, they may become dependent on the internet for social interactions, potentially worsening their reliance on social networking sites in the long term. Therefore, we hypothesize that depression can positively predict problematic SNSU, and problematic SNSU, in turn, can positively predict depression.

Understanding the longitudinal relationship between problematic SNSU and depression is crucial for public health, as it can inform the development of targeted interventions to mitigate the adverse effects of social networking sites on mental health. By elucidating the causal mechanisms and identifying key intervention points, this study aims to contribute to the promotion of healthier and more balanced SNSU, ultimately enhancing mental well-being in the digital age.

## 2 Methods

### 2.1 Participants

The study’s participants were recruited from an elective course at a university in Shandong Province of China, with the incentive of receiving course credits for full participation in the tests. The participants were obligated to complete a series of four questionnaire assessments throughout the semester, spaced approximately one month apart (each interval spanning four weeks). The initial assessment began with 196 participants; however, a small number of students did not attend subsequent sessions for various reasons. Data from participants who did not fully engage in the study were excluded. The final sample consisted of 191 participants who completed all four assessments. This group included 69 males (36.1%) and 122 females (63.9%), with 84 hailing from urban backgrounds (44.0%) and 107 from rural (56.0%). The mean age of the participants was 20.93 years, with a standard deviation of 1.30. This work passed the review of the Institutional Review Board of School of Psychology, SDNU (IRB Number: SDNU2022036). All participants provided their informed consent prior to their inclusion in the study. Due to the nature of the study and the settings in which it was conducted, written consent was not feasible. Therefore, verbal informed consent was obtained from all participants. This process was carefully structured: participants were asked to signify their consent by raising their hand. Similarly, an opportunity to decline participation was also provided, which was indicated in the same manner—by raising their hand. This procedure was meticulously documented and witnessed by two members of the research team to ensure the integrity of the consent process.

### 2.2 Measurement tools

#### 2.2.1 Problematic SNSU

We used the Chinese revised version of the Social Networking Addiction Scale [30] for measurement. The original English scale was developed by Koc and Gulyagci [31]. This scale consists of 8 items, scored on a Likert five-point rating scale ranging from 1 (“completely not true") to 5 (“completely true") aiming to evaluate the degree of social networking addiction. Higher total scores on the scale indicate higher degrees of social networking addiction. The internal consistency reliability (Cronbach’s alpha) of this study ranged from 0.88 to 0.92 at the four points of measurement.

#### 2.2.2 Depression

The Chinese revised version of the Shortened Center for Epidemiologic Studies Depression Scale (CES-D10) [32] was used to measure depression. The original English version was developed by Andresen et al. [33]. This scale involves 10 items, two of which are scored in reverse, rated on a four-point Likert scale ranging from 1 = “none of the time (less than 1 day)" to 4 = “most of the time (5–7 days)”. The higher the total score is, the higher the level of depression. This study measured CES-D10 at four different points, and the Cronbach’s alpha coefficients varied between 0.78 and 0.85.

### 2.3 Data collection procedures and statistical method

The data collection process was conducted in four sessions, with an interval of four weeks between each measurement. The rationale for choosing a four-week interval in our longitudinal study is twofold. First, experimental research has shown that causal relationships can be identified with just a three-week intervention of restricted social media use [24]. Therefore, a four-week interval in a longitudinal study may also capture relationships between variables. Furthermore, existing longitudinal studies have used intervals ranging from six weeks to one year [20,23], suggesting that shorter intervals can more effectively capture dynamic changes between variables. The online testing was facilitated through the Wenjuanxing platform. The measurement of human participants in this study began on September 14, 2022, and concluded on December 7, 2022. Each measurement was conducted among students before their classes, specifically on Wednesday evenings. The researcher first read the instructions to the students, after which the participants accessed the questionnaire by scanning a QR code displayed on the classroom’s main screen. This QR code directed them to the online survey. To ensure data integrity and completeness, the survey platform was designed such that participants could not submit the questionnaire if any questions were left unanswered. The system would prompt them to complete all items, thereby minimizing the risk of missing data.

Descriptive statistics were conducted using SPSS 18.0, and structural equation modeling analysis was performed with AMOS 24.0. In order to delve into the longitudinal relationship between problematic SNSU and depression, we conducted an analysis using structural equation modeling (SEM) on four competing cross-lagged models. Model 1 (M1) acts as a baseline, devoid of cross-lagged paths and encompassing only the autoregressive paths for problematic SNSU and depression, which also include cross-temporal autoregressive paths to improve model fit based on the study of Thompson & Henrich [34], the inter-variable correlations at the first observation (t1), and correlations of residuals at subsequent time points (t2, t3, t4). Model 2 (M2) extends M1 by incorporating cross-lagged paths from problematic SNSU to depression. Model 3 (M3) augments M1 with cross-lagged paths leading from depression to problematic SNSU. Finally, Model 4 (M4) presents an integrative model that compiles all pathways included in the previous models. The four models are shown in S1 Fig.

A good fitting model should meet specific criteria to ensure its adequacy. According to Chen et al. [35] and Baribeau et al. [36], these criteria include a Chi-square degree of freedom ratio (*χ*^2^/df) value of less than 5. Comparative Fit Index (CFI) and Tucker Lewis Index (TLI) values greater than 0.90 indicate acceptable fit, and values greater than 0.95 indicate excellent fit. The Root Mean Square Error of Approximation (RMSEA) value should be less than 0.07 for a good fit, with values less than 0.06 indicating excellent fit. Additionally, the Standardized Root Mean-square Residual (SRMR) value should be less than 0.08 for a good fit, with values less than 0.06 considered excellent.

## 3 Results

### 3.1 Means, standard deviations, and correlation coefficients of variables

The results demonstrated that with the exception of the nonsignificant correlation between problematic SNSU at the initial time point [PSNSU(t1)] and depression measured at all four time points [DEP(t1, t2, t3, t4)], and the nonsignificant correlation between depression at the initial time point [DEP(t1)] and problematic SNSU at the third time point [PSNSU(t3)], all other variables were significantly positively correlated. Detailed results are presented in S1 Table.

### 3.2 Cross-lagged relationships between problematic SNSU and depression

The model comparison is outlined in S2 Table. Notably, no significant differences were found between M2 and M1, M4 and M1, nor between M4 and M3. However, M3 showed superiority over M1, and M4 surpassed M2 in their respective comparisons. Thus, the fitting performance of M4 is not superior to that of M3; hence, according to the principle of parsimony, M3 (*χ*^2^ = 12.35, *df* = 9, *χ*^2^/*df* = 1.37, CFI = 1.00, TLI = 0.99, SRMR = 0.03, RMSEA = 0.04) has been identified as the most robust model among the four under assessment and was further employed to probe into the longitudinal link between problematic SNSU and depression.

The salient findings for Model 3 are visualized in S2 Fig, where only the cross-lagged paths are illustrated to maintain clarity. The results indicate that depression at Time Point 1 can significantly and positively predict problematic SNSU at Time Point 2 (*β* = 0.11, *p* < 0.05). However, depression at Time Point 2 does not significantly predict problematic SNSU at Time Point 3 (*β* = 0.06, *p* = 0.17), nor does depression at Time Point 3 significantly predict problematic SNSU at Time Point 4 (*β* = 0.04, *p* = 0.33).

Additionally, the comparison between M2 and M1 shows no significant differences, indicating that problematic SNSU at an earlier time point does not significantly predict depression at a later time point.

## 4 Discussion

The present study aimed to explore the bidirectional relationship between problematic SNSU and depression across multiple time points. Our findings provide nuanced insights into how these variables interplay over time.

In line with our expectations, depression at Time Point 1 significantly predicted increased problematic SNSU at Time Point 2. This result is consistent with Chen et al. [25], who studied a sample of teachers in mainland China. During our data collection, the COVID-19 pandemic necessitated school closures, mandatory social distancing, and daily nucleic acid testing for students. These measures imposed considerable stress on students’ academic and social lives, leading to increased mental health issues such as depression and anxiety [37,38]. The presence of such mental health issues can lead to problematic SNSU [6]. The theory of compensatory internet use [29] suggests that negative life situations can drive individuals to use the internet to alleviate negative emotions. Social networking sites fulfill this need, compensating for the lack of offline social activities and further contributing to problematic SNSU. This result highlights the potential for college students experiencing depressive symptoms to engage more with social network sites, possibly as a coping mechanism. This finding aligns with previous literature suggesting that individuals with higher levels of depression might turn to social networks for social support, validation, or escape from real-life problems [39–42].

Interestingly, our subsequent analyses revealed that depression at Time Points 2 and 3 did not significantly predict problematic SNSU at later time points (i.e., 3 and 4, respectively). This suggests a diminishing predictive power of depression on problematic SNSU as time progresses, which may indicate the influence of other intervening variables or the possibility that initial increases in problematic SNSU do not sustain over time as a response to depressive symptoms. One reason could be that, according to the theory of compensatory internet use [29], using social networking sites initially meets the social stimulation needs of depressed students, thus improving their mood. Therefore, as the need for social stimulation is satisfied from Time Point 1 to Time Point 2, the motivation for using social media decreases in later stages, reducing the predictive power of depression on problematic SNSU. Another reason is that, just before the second follow-up measurement, a surge in infections in a neighboring city led to the sudden escalation of pandemic control measures at the students’ university. These measures, which remained stringent until the end of the fourth measurement, heightened students’ reliance on social media for receiving school information, reporting personal data, attending online classes, and communicating with teachers and classmates. This strong policy intervention may have weakened the predictive power of depression on problematic SNSU.

Additionally, our model comparison analysis revealed that problematic SNSU at an earlier time point did not significantly predict later depression. This result was inconsistent with our expectations and two existing longitudinal studies [3,21]. These studies, conducted among university students in Hong Kong and Taiwan, found that problematic SNSU significantly predicted levels of depression. The inconsistency might be attributed to the stringent COVID-19 measures in mainland China, where strict dynamic zero-COVID policies, school closures, and mandatory testing were implemented. In such an environment, social media became integral to students’ daily academic and social activities, making excessive use of social networking sites a norm during the COVID-19 pandemic. Excessive use of social media, contrary to causing depression, became a means for students to maintain their studies and daily lives. Chen et al. [25] found similar results in a longitudinal study of teachers in mainland China, further supporting the influence of government policies during the COVID-19 pandemic.

Additionally, it is noteworthy that most international longitudinal studies on the relationship between problematic SNSU and depression found unidirectional predictive relationships. Some studies identified problematic SNSU as predicting depression [3,21–23], while others found depression predicting problematic SNSU [25,26]. We identified only one study [27] that found a bidirectional predictive relationship, but this study used two sub-samples—one examining problematic SNSU predicting depression and the other examining depression predicting problematic SNSU. This design might not accurately capture the bidirectional relationship. Therefore, we can conclude that existing studies do not consistently show a bidirectional predictive relationship between problematic SNSU and depression, with more studies supporting a unidirectional relationship. It is essential to interpret these results cautiously, as the lack of bidirectional influence might be due to research design limitations and the asynchronous temporal effects between the two variables. Longitudinal studies typically measure both variables simultaneously at multiple time points. If the effect of problematic SNSU on depression takes longer to manifest than the effect of depression on problematic SNSU, longitudinal studies may capture the effect of depression on problematic SNSU but not vice versa, and vice versa. Future research should adopt new designs to capture this bidirectional relationship more effectively.

Based on our study, several practical implications arise for addressing problematic SNSU and its relationship with depression among college students. It is crucial for mental health professionals and educators to recognize that depression can predict an increase in problematic SNSU over time, highlighting the importance of early identification and intervention for students with depressive symptoms. Timely mental health support can mitigate the rise of problematic SNSU. Additionally, the diminishing predictive power of depression on SNSU over time suggests that initial increases in SNSU may serve as a coping mechanism that loses effectiveness, indicating the need for sustainable coping strategies beyond social media. Mental health programs should equip students with alternative methods for managing stress and maintaining social connections, particularly during heightened stress periods like the COVID-19 pandemic. Our findings also emphasize the impact of external factors, such as stringent COVID-19 measures, on the depression-SNSU relationship, suggesting that interventions should adapt to varying circumstances. While our study did not find a significant predictive effect of problematic SNSU on subsequent depression, future research should use innovative designs to capture this relationship’s temporal dynamics more accurately. Understanding these complex interactions is crucial for developing effective interventions to support the mental health and well-being of college students in an increasingly digital world.

Several limitations of our study warrant careful consideration. First, the data collection relied solely on self-reported measures, which may be subject to biases such as social desirability and recall inaccuracies. Future studies should incorporate objective measures or third-party reports to enhance data reliability and validity. Second, our sample consisted exclusively of college students, limiting the generalizability of our findings to other populations. Broader studies including diverse age groups, educational backgrounds, and cultural contexts are needed to confirm the robustness of these results. Third, our study did not account for potential mediating or moderating variables that could influence the relationship between problematic SNSU and depression. Factors such as personality traits, social support, and specific coping mechanisms might play significant roles and should be examined in future research to provide a more comprehensive understanding of these dynamics. Additionally, the stringent COVID-19 measures during data collection may have uniquely influenced the observed relationships, suggesting the need for studies in different contexts to assess the consistency of these findings. Lastly, the temporal dynamics and bidirectional nature of the relationship between problematic SNSU and depression require further investigation with innovative longitudinal designs to capture these complex interactions more accurately. Addressing these limitations will be crucial for developing effective interventions and supporting the mental health of college students in an increasingly digital world.

## 5 Conclusion

This longitudinal study provides nuanced insights into the relationship between problematic SNSU and depression among college students. Our findings indicate that depression at an earlier time point significantly predicts later increased problematic SNSU, consistent with the theory of compensatory internet use. However, this predictive power diminishes over time, suggesting that initial SNSU increases as a coping mechanism may lose effectiveness. Interestingly, problematic SNSU did not predict later depression, diverging from previous studies. This discrepancy may be influenced by the unique pandemic context, where stringent measures heightened reliance on social media. Our results underscore the importance of early mental health interventions and sustainable coping strategies beyond social media. Future research should address our study’s limitations by incorporating objective measures, diverse populations, and examining mediating variables. Understanding these complex interactions is vital for developing effective interventions to support college students’ mental health in an increasingly digital world.

## Supporting information

S1 Fig Four Hypothetical cross-lagged panel models of social networking sites use and depression. (TIF)

S2 Fig The Results of cross-lagged model of social networking sites use and depression. (TIF)

S1 Table Descriptive statistics and correlation analysis of problematic SNSU and depression. (DOCX)

S2 Table Comparison of fit index of each model. (DOCX)
