# Supplementary figures and images for: Exploring the relationship between problematic social networking sites use and depression: A longitudinal study
Source: PLoS One. 2024 Nov 18;19(11):e0313362. doi: 10.1371/journal.pone.0313362 (PMC11573223; doi:10.1371/journal.pone.0313362)

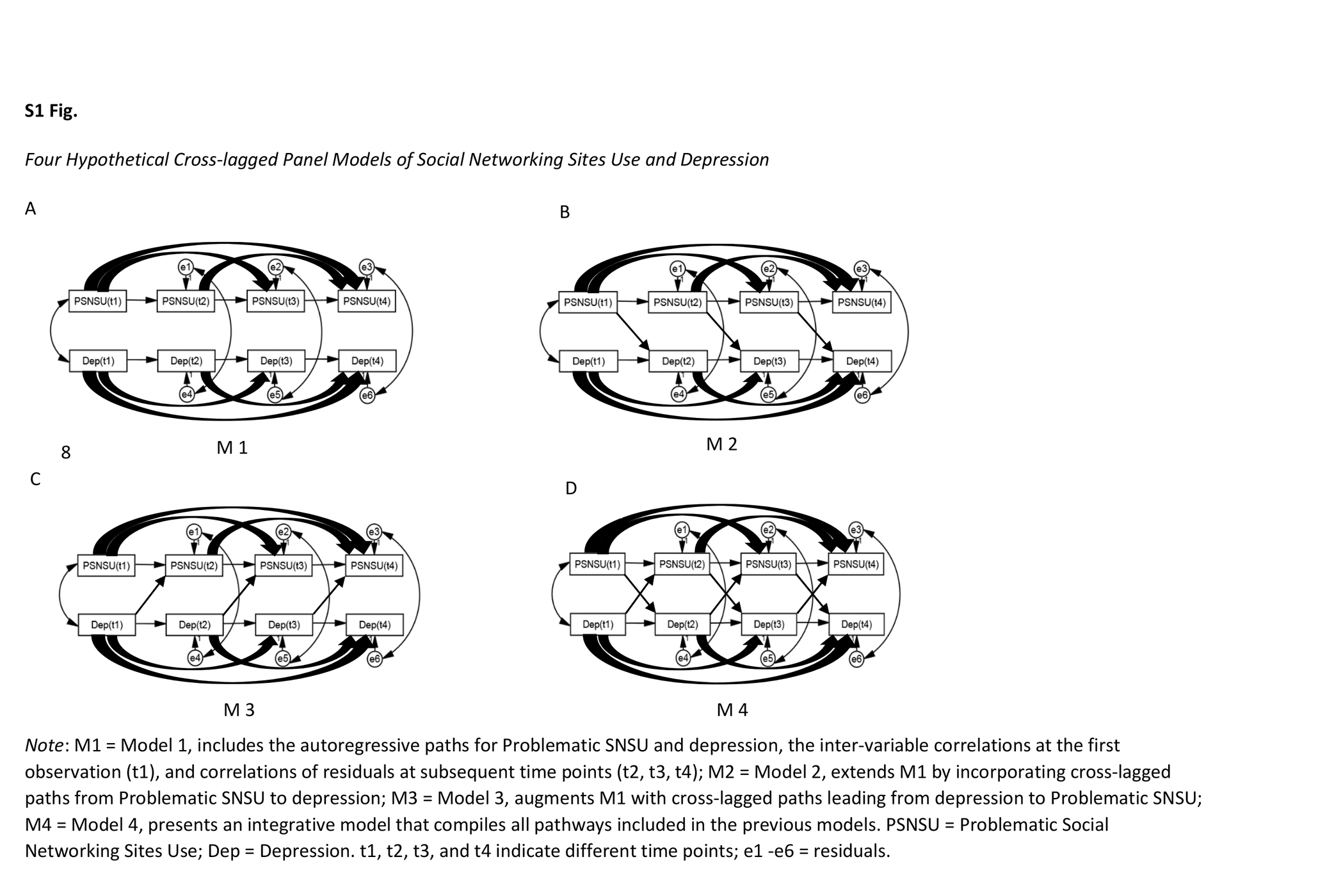

Supplement: S1 Fig — (TIF) [file pone.0313362.s001.tif]

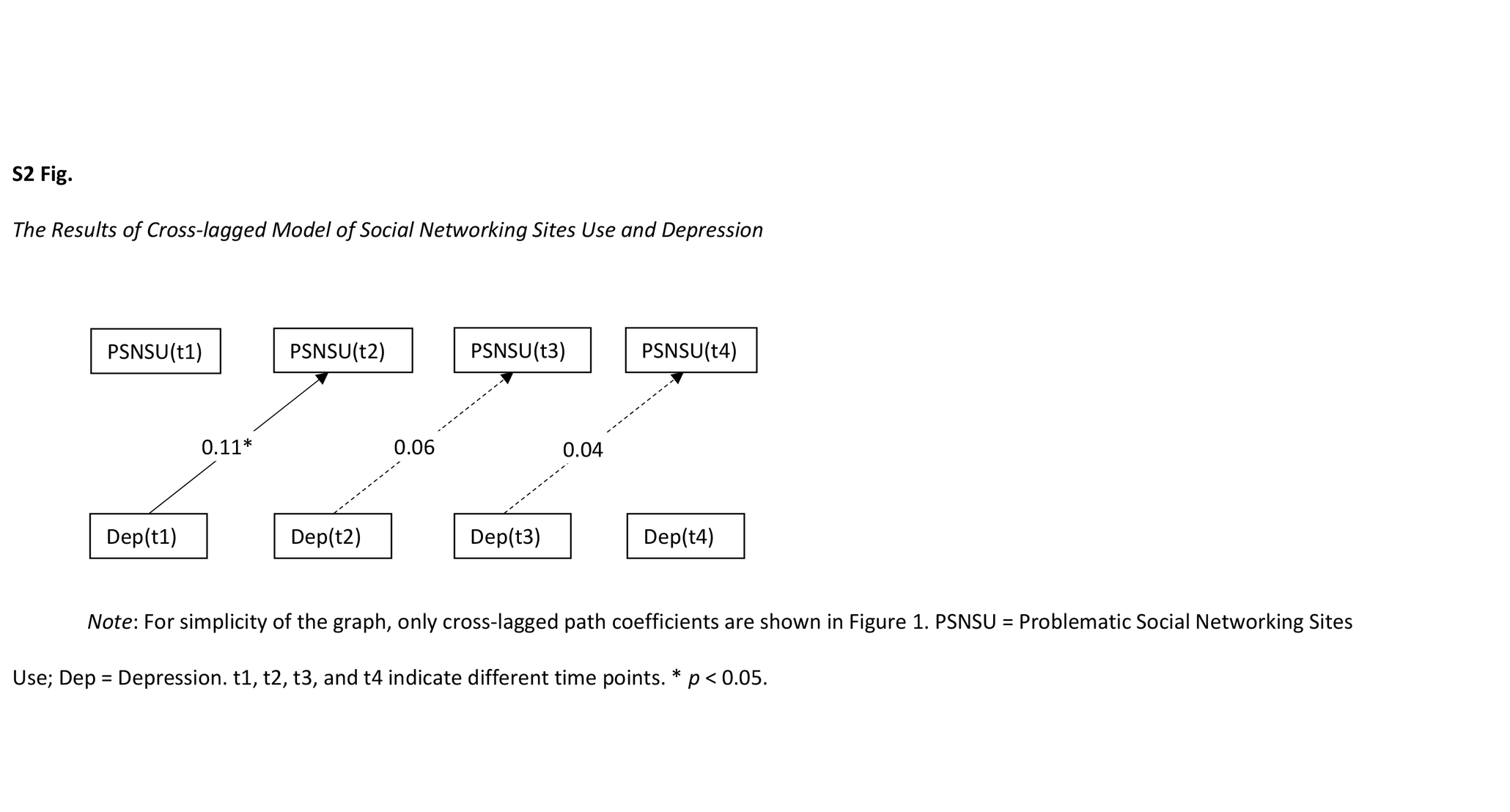

Supplement: S2 Fig — (TIF) [file pone.0313362.s002.tif]
