# Supplementary material for: Exploring the relationship between problematic social networking sites use and depression: A longitudinal study
Source: PLoS One. 2024 Nov 18;19(11):e0313362. doi: 10.1371/journal.pone.0313362 (PMC11573223; doi:10.1371/journal.pone.0313362)
Supplement: S1 Table — (DOCX) [file pone.0313362.s003.docx]

**S1 Table**

*Descriptive Statistics and Correlation Analysis of Problematic SNSU and Depression*

|  | 1 | 2 | 3 | 4 | 5 | 6 | 7 | 8 |
| --- | --- | --- | --- | --- | --- | --- | --- | --- |
| 1.PSNSU (t1) | — |  |  |  |  |  |  |  |
| 2.DEP (t1) | 0.125 | — |  |  |  |  |  |  |
| 3.PSNSU (t2) | 0.735** | 0.201** | — |  |  |  |  |  |
| 4.DEP (t2) | 0.088 | 0.630** | 0.182* | — |  |  |  |  |
| 5.PSNSU (t3) | 0.719** | 0.123 | 0.787** | 0.184* | — |  |  |  |
| 6.DEP (t3) | 0.123 | 0.521** | 0.194** | 0.608** | 0.214** | — |  |  |
| 7.PSNSU (t4) | 0.751** | 0.155* | 0.796** | 0.190** | 0.755** | 0.197** | — |  |
| 8.DEP (t4) | 0.053 | 0.483** | 0.166* | 0.647** | 0.213** | 0.645** | 0.224** | — |
| *M±SD* | 2.47±0.82 | 1.66±0.47 | 2.52±0.78 | 1.68±0.46 | 2.55±0.85 | 1.69±0.51 | 2.61±0.83 | 1.70±0.51 |

*Note*: PSNSU=Problematic SNSU; DEP=Depression. t1-t4 represents different points in time. *p<0.05; **p<0.01. The interval between every two measurements is four weeks.
