# Supplementary material for: Exploring the relationship between problematic social networking sites use and depression: A longitudinal study
Source: PLoS One. 2024 Nov 18;19(11):e0313362. doi: 10.1371/journal.pone.0313362 (PMC11573223; doi:10.1371/journal.pone.0313362)
Supplement: S2 Table — (DOCX) [file pone.0313362.s004.docx]

**S2 Table**

*Comparison of Fit Index of Each Model*

|  | Fit index of each model | | | | | |  | Model comparison | | |
| --- | --- | --- | --- | --- | --- | --- | --- | --- | --- | --- |
|  | χ^2^ | *df* | CFI | TLI | SRMR | RMSEA |  | Δχ^2^ | Δ*df* | *p* |
| M1 | 20.19 | 12 | 0.99 | 0.98 | 0.06 | 0.06 | M2 vs. M1 | 2.71 | 3 | 0.44 |
| M2 | 17.48 | 9 | 0.99 | 0.97 | 0.04 | 0.07 | M3 vs. M1 | 7.84 | 3 | < 0.05 |
| M3 | 12.35 | 9 | 1.00 | 0.99 | 0.03 | 0.04 | M4 vs. M1 | 10.40 | 6 | 0.11 |
| M4 | 9.79 | 6 | 1.00 | 0.98 | 0.02 | 0.06 | M4 vs. M2 | 7.69 | 3 | 0.05 |
|  |  |  |  |  |  |  | M4 vs. M3 | 2.56 | 1 | 0.11 |

*Note*: M1 = Model 1, includes the autoregressive paths for Problematic SNSU and depression, the inter-variable correlations at the first observation (t1), and correlations of residuals at subsequent time points (t2, t3, t4); M2 = Model 2, extends M1 by incorporating cross-lagged paths from Problematic SNSU to depression; M3 = Model 3, augments M1 with cross-lagged paths leading from depression to Problematic SNSU; M4 = Model 4, presents an integrative model that compiles all pathways included in the previous models. *χ*^2^ = Chi-square; df = degree of freedom; CFI = comparative fit index; TLI = Tucker Lewis index; SRMR = standardized root mean-square residual; RMSEA = root mean square error of approximation.
